# Prediction of Mature Body Weight of Indigenous Camel (*Camelus dromedarius*) Breeds of Pakistan Using Data Mining Methods

**DOI:** 10.3390/ani15142051

**Published:** 2025-07-11

**Authors:** Daniel Zaborski, Wilhelm Grzesiak, Abdul Fatih, Asim Faraz, Mohammad Masood Tariq, Irfan Shahzad Sheikh, Abdul Waheed, Asad Ullah, Illahi Bakhsh Marghazani, Muhammad Zahid Mustafa, Cem Tırınk, Senol Celik, Olha Stadnytska, Oleh Klym

**Affiliations:** 1 Laboratory of Biostatistics, Bioinformatics and Animal Research, West Pomeranian University of Technology, 71-270 Szczecin, Poland; 2 Centre for Advanced Studies in Vaccinology and Biotechnology (CASVAB), University of Balochistan, Quetta 87300, Pakistan; 3 Department of Livestock and Poultry Production, Bahauddin Zakariya University, Multan 60800, Pakistan; 4 Department of Animal Nutrition, Lasbela University of Agriculture, Water and Marine Sciences, Uthal 90150, Pakistan; 5 Biometry and Genetics Unit, Department of Animal Science, Agricultural Faculty, Iğdır University, 76000 Iğdır, Turkey; 6 Biometry and Genetics Unit, Department of Animal Science, Agricultural Faculty, Bingol University, 12000 Bingol, Turkey; 7 Institute of Agriculture in the Carpathian Region of the National Academy of Agrarian Sciences of Ukraine, 81115 Obroshyne, Ukraine

**Keywords:** mature body weight, productive traits, reproductive traits, data mining, prediction

## Abstract

The determination of the live body weight of camels (required for their successful breeding) is a rather difficult task due to the problems with handling and restraining these animals. Therefore, the main aim of this study was to predict the ABW of eight indigenous camel (*Camelus dromedarius*) breeds of Pakistan (Bravhi, Kachi, Kharani, Kohi, Lassi, Makrani, Pishin, and Rodbari). Selected productive (hair production, milk yield per lactation, and lactation length) and reproductive (age of puberty, age at first breeding, gestation period, dry period, and calving interval) traits served as the predictors. Six data mining methods [classification and regression trees (CARTs), chi-square automatic interaction detector (CHAID), exhaustive CHAID (EXCHAID), multivariate adaptive regression splines (MARSs), MLP, and RBF] were applied for ABW prediction. Additionally, hierarchical cluster analysis with Euclidean distance was performed for the phenotypic characterization of the camel breeds. The highest Pearson correlation coefficient between the observed and predicted values (0.84, *p* < 0.05) was obtained for MLP, which was also characterized by the lowest root-mean-square error (RMSE) (20.86 kg), standard deviation ratio (SD_ratio_) (0.54), mean absolute percentage error (MAPE) (2.44%), and mean absolute deviation (MAD) (16.45 kg). The most influential predictor for all the models was the camel breed. The applied methods allowed for the moderately accurate prediction of ABW (average R^2^ equal to 65.0%) and the identification of the most important productive and reproductive traits affecting its value. However, one important limitation of the present study is its relatively small dataset, especially for training the ANN (MLP and RBF). Hence, the obtained preliminary results should be validated on larger datasets in the future.

## Simple Summary

Camel (*Camelus dromedarius*) is an important animal species that has the ability to survive for a long time under arid, semi-arid, and harsh desert environmental conditions with a scarcity of water and feed. There are increasing demands for their meat, milk, hair, and hides in the developing rural economies of countries like Pakistan and Bangladesh. The determination of the live body weight of camels is a rather difficult task due to the problems with handling and restraining these animals. Consequently, its prediction has received considerable attention for estimating feed amounts, market prices, and providing veterinary care. In our study, the adult body weight (ABW) of eight indigenous camel breeds of Pakistan was predicted using selected machine learning algorithms. The most accurate results were obtained for the artificial neural network (ANN), and the predicted values were similar to the observed ones. In addition, camel breed was the most influential factor affecting ABW. Consequently, the applied methods allowed for the moderately accurate prediction of the ABW of camels and the identification of the most important productive and reproductive traits determining its value. However, one important limitation of the present study is its relatively small dataset, especially for training the ANN [multilayer perceptron (MLP) and radial basis function networks (RBFs)]. Hence, the obtained preliminary results should be validated on larger datasets in the future.

## 1. Introduction

Camel (*Camelus dromedarius*) is a multipurpose animal species particularly adapted to hot and arid environments such as deserts [1]. It provides milk, meat, wool, hair, and hides, playing a major role in the developing rural economies of countries like Pakistan [2] and Bangladesh [3]. Camels are also used for riding and carrying goods in agricultural operations. They are versatile animals capable of converting scanty plant material into milk and meat [1]. In comparison to other domestic species, camels eat less, sleep in shorter intervals, and have a longer-lasting memory [4,5,6]. They also yield milk in all seasons, including dry periods, being an ideal option for pastoralists. Therefore, further studies on their phenotypic characterization are of great importance for breeding sustainability and gene source conservation [7].

The determination of the live body weight of camels is a rather difficult task due to the problems with their handling and restraining during phenotypic measurements. This, in turn, results from their wild nature and large body size (especially in a mature age) [8,9]. Consequently, the prediction of ABW has received considerable attention for estimating feed amounts, market prices, and providing veterinary care in breeding practice [10]. It is especially useful in rural and pastoral production systems, where weighbridges are unavailable [7]. Different methods, such as weighing tapes, visual appraisal, linear body measurements [11], and digital image processing [12], emerged as alternative procedures for body weight determination in large animals. Significant correlations between body size and weight can be used for estimating the latter via mathematical equations [13,14], e.g., live weight = shoulder height × chest girth × hump girth × 50. Many studies showed that chest circumference, body length, hip width, and shoulder height are the most reliable parameters for the live weight assessment of domestic animals [15,16,17,18,19,20].

The morphological traits employed in body weight prediction are essential for the identification and discrimination of breeds [1]. They are used to describe breed standards and conserve indigenous gene sources as indirect selection criteria, thus making it possible to obtain elite camel herds with the support of molecular characterization in the future [7].

Most studies on ABW prediction have used zoometric measurements so far [7,9,10,21,22,23]. However, in the absence of such data, other available variables could be utilized. One group of such predictors includes productive and reproductive parameters, influencing the revenue and profit of camel breeders. For instance, Knoess [24,25] and Knoess et al. [26] found an association between milk yield and ABW. Productive and reproductive performance, controlled by genetic and non-genetic factors, affects the sustainability of camel breeding systems in Pakistan, Saudi Arabia, Bangladesh, and other countries [27]. Statistical methods may facilitate our understanding of the relationship between productive and reproductive traits and ABW, which would allow for breeders to increase their profit per animal. Such methods include data mining algorithms [e.g., multivariate adaptive regression splines (MARSs)], some of which have already been applied to body weight prediction in camels [7,21]. However, the literature lacks their comprehensive comparison in this context.

Therefore, the aim of the present study was to predict the ABW of indigenous Pakistani camels based on the available productive and reproductive traits using selected data mining methods [classification and regression trees (CARTs) [28], chi-square automatic interaction detection (CHAID) [29], MARS [7], multilayer perceptrons (MLP) [30], and the radial basis function network (RBF) [30].

## 2. Materials and Methods

### 2.1. Location, Management and Data Collection

The data were retrospectively collected from the Quetta, Kharan, Makran, and Kalat farms of Balochistan (latitude: 28.4907°; longitude: 65.0958°; 769.9 m above sea level) located in Pakistan. Average annual temperature and precipitation in the study region were 23 °C and 213 mm, respectively.

### 2.2. Quantitative and Qualitative Traits

ABW (predicted variable), productive traits (hair production, milk yield per lactation, and lactation length) and reproductive traits (age of puberty, age at first breeding, gestation length, dry period, and calving interval) were recorded from eight indigenous camel breeds (Bravhi, Kachi, Kharani, Kohi, Lassi, Makrani, Pishin, and Rodbari) raised under Pakistani conditions. ABW was determined with an electronic scale in the morning on an empty stomach, so that the accuracy and comparability of the measurements were assured. Descriptive statistics and Pearson’s correlation coefficients for the predictor and predicted variables are presented in Table 1, Table 2 and Table 3.

The full dataset of ABW records was randomly divided into three subsets: a training set (used for model building; approximately 50% of the whole dataset; 70 records), a validation set (utilized to prevent overfitting; approximately 25% of the entire dataset; 30 records), and a test set (used for model predictive performance evaluation; approximately 25% of the whole dataset; 35 records). In the cases of MLR, CART, CHAID, exhaustive CHAID (EXCHAID) and MARS, the validation set was incorporated into the training set (approximately 75% of the entire dataset; 100 records). Due to the small training sample size, a second performance evaluation method (i.e., 10-fold cross-validation) was also applied [29].

### 2.3. Statistical Analysis

The Kruskal–Wallis analysis of variance was performed for all the variables among the breeds. The first method for ABW prediction was CART [31]. Splitting based on the least-squared deviation and pruning according to variance (as a stopping rule) were performed in the tree construction procedure. The minimum node size of 13 was adopted as an additional stopping criterion [32]. Moreover, 10-fold cross-validation with a one-standard error rule was applied in order to find the most effective regression tree with appropriate complexity and fit to the training data. The second and third tree-based algorithms used in the present study were CHAID [33] and EXCHAID (in the exhaustive mode), with the value of the F test as a splitting criterion. A minimal node size of 13 and a *p*-value for splitting equal to 0.05 served as the stopping criteria [32]. In addition, the Bonferroni adjustment was utilized to correct for the *p*-values of the best predictor at each split, whereas 10-fold cross-validation was used to prevent over-fitting. In all the tree-based methods, relative predictor importance was calculated by summing—over all nodes in the tree—the drop (delta) in the resubstitution estimate [delta(R)] and expressing these sums relative to the largest sum found over all predictors (the most important variable).

For the next method, the following MARS model was applied in the current study [34]:

(1) $$\hat{y}=\beta_{0}+\sum_{m=1}^{M}\beta_{m}\prod_{k=1}^{K_{m}}h_{km}\left(X_{v(k,m)}\right),$$

where *ŷ* is the predicted value of the dependent variable; *β_0_* is the constant; *β_m_* is the coefficient of the *m*th basis function; *h_km_*(*X_v_*_(*k,m*)_) is the basis function, in which *v*(*k*,*m*) is the index of the predictor used in the *m*th component of the *k*th product; and *K_m_* is the parameter limiting the order of interaction.

The maximum number of basis functions was 21, and no interactions were allowed. After building the most complex additive MARS model, the basis functions that were associated with the smallest increase in goodness-of-fit were removed in the process of so-called pruning based on the following generalized cross-validation error (*GCV*) [35]:

(2) $$GCV\left(\lambda \right)=\frac{\sum_{i=1}^{n}{\left(y_{i}-{\hat{y}}_{i}\right)}^{2}}{{\left\lceil 1-\frac{M\left(\lambda \right)}{n}\right\rceil }^{2}},$$

where *n* is the number of training cases, *y_i_* is the observed value of the dependent variable, *ŷ_i_* is the predicted value of the dependent variable, and *M*(*λ*) is the penalty function for the complexity of the model containing *λ* terms.

The model with the smallest *GCV* was considered as the best one. The relative predictor importance was based on the number of references to each of them by the final MARS model.

Lastly, two types of ANN were applied: MLP and RBF. In the first stage of the RBF training, the location and radial spread of the basis functions were fixed using the input data. In the second stage, the weights connecting the radial functions to the output neurons were determined [36]. MLP was trained with the Broyden–Fletcher–Goldfarb–Shanno algorithm [37]. The data mining module of the Statistica computer program enabled the automatic selection of the optimal network architecture and parameters (the number of neurons in the hidden layer, the type of postsynaptic potential and activation functions, the number of training epochs, etc.). All the networks were trained until reaching the highest Pearson correlation coefficient on the validation set (part of the whole dataset utilized to prevent overfitting). Relative predictor importance was based on the sum of squared residuals for the network with the removed predictor. Subsequently, the predictors were sorted according to the ratios (the sum of squared residuals for the full model relative to the model with the removed predictor).

For the sake of meaningful comparison, multiple linear regression (MLR) was also applied as a more traditional statistical method, in which predictors were ranked according to their *p*-values. During the model development process, all the assumptions of MLR were verified (the lack of collinearity and autocorrelation, homoscedasticity, and the normal distribution of residuals).

The predictive performance of all the models was evaluated on the independent test set with the following goodness-of-fit criteria [28,29,30]:

1. Pearson correlation coefficient (*r*) between the observed and predicted values;
2. Coefficient of determination (*R*^2^):

(3) $$R^{2}=1-\frac{\sum_{i=1}^{n}{\left(y_{i}-{\hat{y}}_{i}\right)}^{2}}{\sum_{i=1}^{n}{\left(y_{i}-\bar{y}\right)}^{2}},$$

3. Akaike information criterion (*AIC*):

(4) $$\left\{\begin{array}{cc} AIC=n\cdot ln\left[\frac{1}{n}\sum_{i=1}^{n}{\left(y_{i}-{\hat{y}}_{i}\right)}^{2}\right]+2k, & if\;n/k>40\;\\ {AIC}_{c}=AIC+\frac{2k(k+1)}{n-k-1} & otherwise \end{array}\right.,$$

4. Root-mean-square error (*RMSE*):

(5) $$RMSE=\sqrt{\frac{1}{n}\sum_{i=1}^{n}{\left(y_{i}-{\hat{y}}_{i}\right)}^{2}},$$

5. Mean error (*ME*):

(6) $$ME=\frac{1}{n}\sum_{i=1}^{n}\left(y_{i}-{\hat{y}}_{i}\right),$$

6. Mean absolute deviation (*MAD*):

(7) $$MAD=\frac{1}{n}\sum_{i=1}^{n}\left|y_{i}-{\hat{y}}_{i}\right|,$$

7. Standard deviation ratio (*SD_ratio_*):

(8) $${SD}_{ratio}=\frac{S_{m}}{S_{d}},$$

8. Global relative approximation error (*RAE*):

(9) $$RAE=\sqrt{\frac{\sum_{i=1}^{n}{\left(y_{i}-{\hat{y}}_{i}\right)}^{2}}{\sum_{i=1}^{n}{y_{i}}^{2}}},$$

9. Mean absolute percentage error (*MAPE*):

(10) $$MAPE=\frac{1}{n}\sum_{i=1}^{n}\left|\frac{y_{i}-{\hat{y}}_{i}}{y_{i}}\right|\cdot 100,$$

where *n* is the training sample size, *k* is the number of model parameters, *y_i_* is the real value of the dependent variable (ABW), ${\hat{y}}_{i}$ is the predicted value of the dependent variable, $\bar{y}$ is the mean value of the dependent variable, *s_m_* is the standard deviation of the model errors, and *s_d_* is the standard deviation of the dependent variable.

The final stage of the present study involved hierarchical cluster analysis (agglomerative method with single linkage and Euclidean distance) based on all the cases (*n* = 135) and all the variables (ABW and continuous predictors) both for the individual animals and breeds. The construction, training, and testing of the ANN, the development of the tree and MARS models, and hierarchical cluster analysis were carried out using the Statistica program (v. 13.3, Tibco Inc., Tulsa, OK, USA). For the calculation of the predictive performance measures, the ehaGoF R package (version 0.1.1) was used (R Development Core Team 2020, R Foundation for Statistical Computing, Vienna, Austria). Statistical significance was considered at *p* < 0.05.

## 3. Results

In general, the continuous predictors were weakly or moderately (although sometimes significantly) correlated with ABW (Table 3). However, significant differences among the breeds occurred for most variables (Table 4). In particular, the lowest ABW (584.5 kg) was observed for Lassi, and the highest one (703.7 kg) was recorded for Pishin (*p* < 0.05).

**Figure 1:**
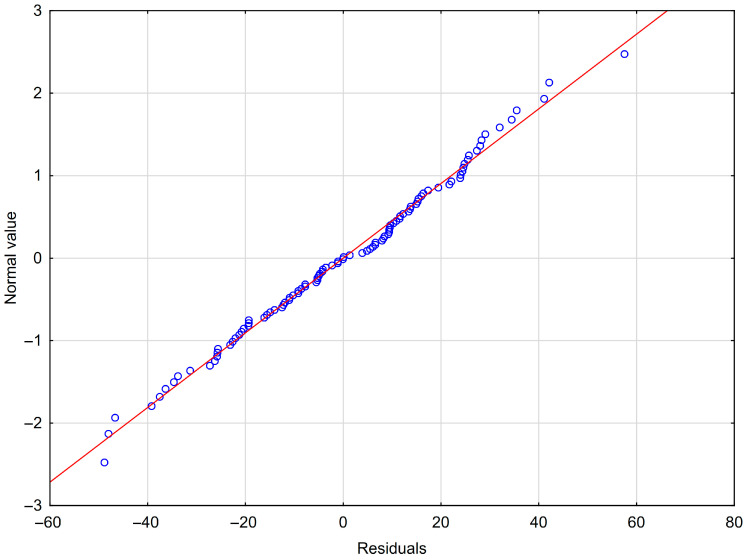
The residual normality plot for MLR.

The following MLR model was obtained in our study: ABW = 681.94 − 1.73 × HAIR + 0.05 × MILK − 0.07 × LACT + 0.04 × AGEP − 0.08 × AGEB − 0.08 × GEST − 0.27 × DP + 0.15 × CI − 28.91 × BREED_Kachi + 8.31 × BREED_Pishin − 5.13 × BREED_Makrani − 16.19 × BREED_Kohi − 70.85 × BREED_Lassi − 13.14 × BREED_Rodbari + 43.95 × BREED_Kharani. A residual normality plot is shown in Figure 1. All the other MLR assumptions were fulfilled (Shapiro–Wilk *W* = 0.99, *p* = 0.7983; *r* = −0.13, *p* = 0.2138).

**Figure 2:**
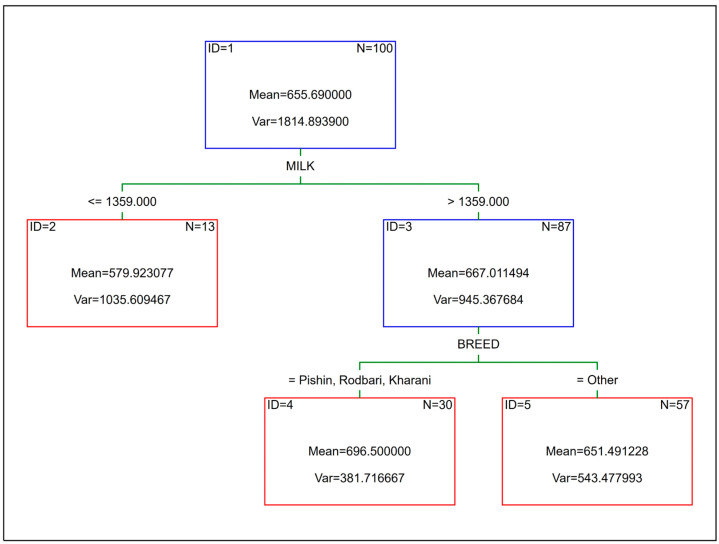
CART for ABW (kg) of camels.

**Figure 3:**
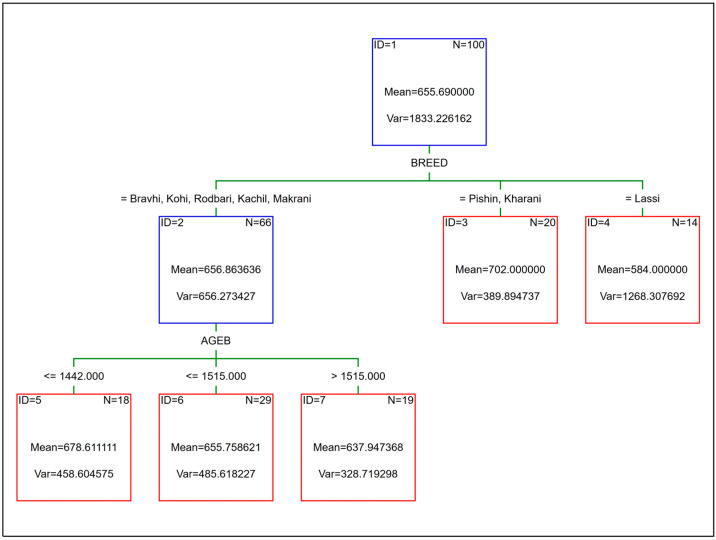
CHAID and EXCHAID trees for ABW (kg) of camels.

The layout of the CART, CHAID, and EXCHAID trees is shown in Figure 2 and Figure 3, respectively (the last two were the same). The first node (ID = 1) in the CART, called the root node, contained all the training cases (*n* = 100), which were subsequently divided into two smaller subsets (the so-called child nodes) based on the value of the MILK variable (the mean and the Var in each node denote the average ABW and its variance, respectively, for all the cases within that node). The left child node (ID = 2) was the leaf node at the same time, since no more splits were made in this case. The right child node (ID = 3) was further divided into two leaf nodes (ID = 4 and ID = 5) based on the camel breed (the BREED variable). A similar split of the root node was observed for the CHAID and EXCHAID trees; however, the first division of the whole training set (*n* = 100) was based on the BREED variable instead of MILK. The two right child nodes (ID = 3 and ID = 4) were the leaf nodes as no additional splits were performed in this case, whereas the left child node (ID = 2) was further divided into three leaf nodes (ID = 5, ID = 6 and ID = 7) based on the value of the AGEB variable. In order to predict ABW from the CART, CHAID, and EXCHAID trees, it was necessary to move from the top of the tree (the root node) to the bottom (the leaf nodes), taking into account the values of individual predictors at each stage. The final ABW is the mean of the selected leaf node.

The resulting MARS model (including six basis functions) is given by the following formula: ABW = 709.83 − 0.20 × max(0, 1690.00 − MILK) − 0.29 × max(0, DP − 325.00) − 0.55 × max(0, AGEB − 1382.00) + 54.09 × max(0, BREED_Kharani) + 0.42 × max(0, AGEB − 1453.00) − 0.08 × max(0, 1264.00 − AGEP). The best MLP and RBF networks had 16-10-1 and 16-17-1 architectures, respectively (the number of neurons in the input, hidden, and output layers, respectively). Exponential and hyperbolic tangent activation functions were used in the hidden and output MLP layers, respectively. The Broyden–Fletcher–Goldfarb–Shanno algorithm took six epochs to converge.

**Figure 4:**
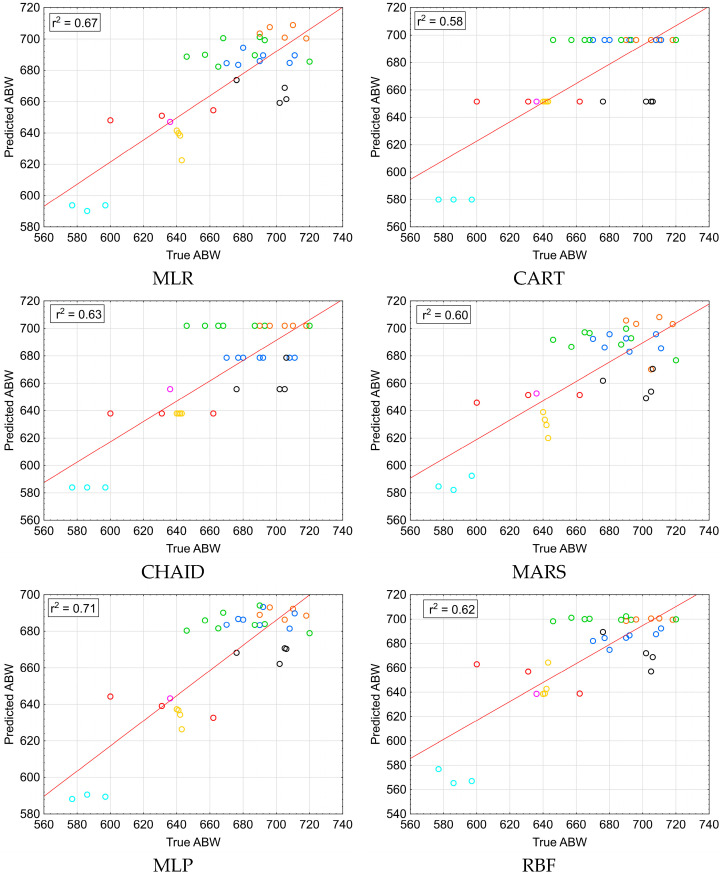
Observed vs. predicted ABW (kg) for individual models and breeds. Bravhi—yellow; Kachi—red; Pishin—orange; Makrani—black; Kohi—violet; Lassi—turquoise; Rodbari—blue; Kharani—green.

The basic predictive performance measures for all the models are presented in Table 5 and Figure 4. Since the results of the 10-fold cross-validation were, in general, the same as for the train–test split procedure, the latter is presented in our study. It can be seen that the highest Pearson correlation coefficient between the observed and predicted values was found for MLP. It was slightly higher than those for CHAID, EXCHAID, RBF, and MARS. It should be noted that the second highest value of the correlation coefficient was characteristic of MLR; however, the differences among the correlation coefficients were not statistically significant. MLP was also characterized by the lowest *RMSE*, *SD_ratio_*, *MAPE*, and *MAD*, while the smallest absolute values of *ME* were obtained for CHAID and EXCHAID. *ME* was positive for all the models, except for MLR, CART, and RBF, which means that almost all of them overestimated ABW. *RAE* was null in all the cases, whereas the standard and corrected *AIC* were lowest for MLP. However, one should take into account that the models developed in the present study had very different numbers of parameters and their *AIC* values are not directly comparable.

The analysis of relative predictor importance (Table 6) showed that the most influential factor affecting ABW for MLR, CART, CHAID, EXCHAID, MLP, and RBF was BREED. Only in the case of MARS, it was ranked lower (the second position). For MARS, the most important predictor was age at first breeding (AGEB), which was also influential for MLR, CART (the third position), and to a lesser extent for MLP, CHAID, and EXCHAID (the fourth or fifth position). However, this variable was not so important for RBF (the ninth position). The third most influential predictor for almost all the models was milk yield per lactation (MILK) (the second position for CART, MARS, and MLP and the third position for CHAID, EXCHAID, and RBF). Only in the case of MLR, it was ranked lower (the sixth position). The order of relative predictor importance for the remaining variables differed among the models.

**Figure 5:**
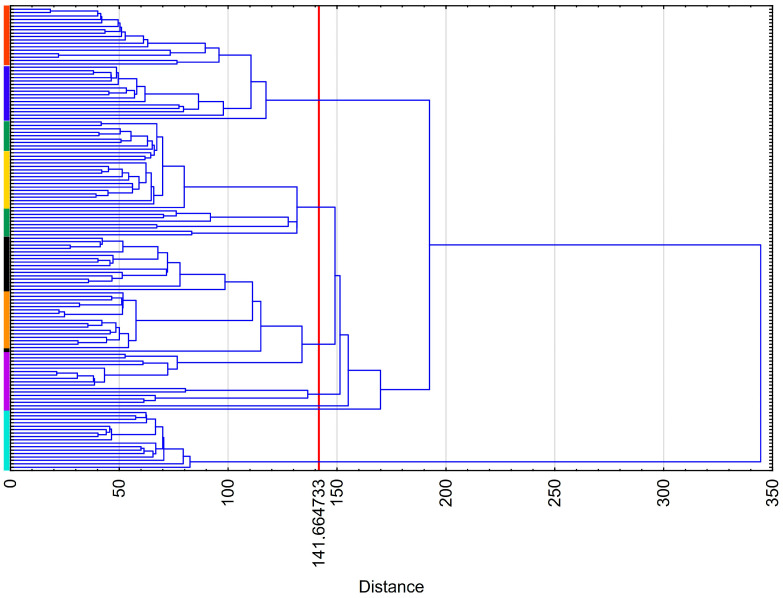
Dendrogram for camels (Bravhi—red; Kharani—blue; Kachi—green; Makrani—yellow; Pishin—black; Rodbari—orange; Kohi—purple; Lassi—cyan).

**Figure 6:**
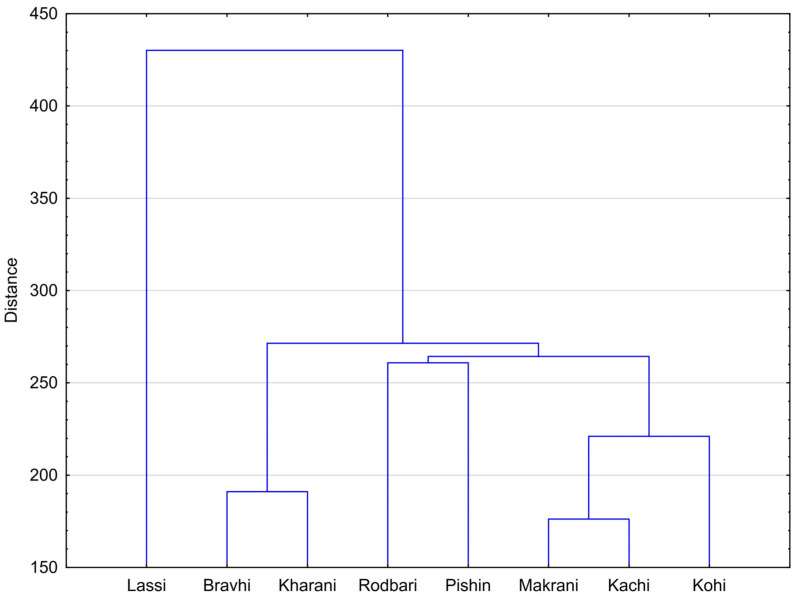
Dendrogram for camel breeds.

Finally, the results of hierarchical cluster analysis (a dendrogram) are shown in Figure 5 and Figure 6. It can be seen that the number of clusters (seven) corresponded to the number of camel breeds (Figure 5). Moreover, Lassi was the most distant cluster. The remaining three groups of similar breeds included Bravhi and Kharani (group 1); Rodbari and Pishin (group 2); and Makrani, Kachi, and Kohi (group 3).

## 4. Discussion

A moderate correlation between the predictors and ABW affected the final predictive performance. Consequently, ABW prediction was moderately accurate. The *R*^2^ values ranged between 0.58 and 0.71 (0.66 on average), which means that about 29–42% of the variance in ABW was not accounted for by the models developed in our study. The inclusion of other more strongly correlated variables would improve the model predictive performance, and thus the ABW prediction accuracy. The best model for ABW prediction in camels was MLP, followed by MLR (according to Pearson’s correlation coefficient between the observed and predicted values). However, the differences in the correlation coefficients were not statistically significant, although some numerical fluctuations could be observed. The relatively strong predictive performance of MLR (compared to the other models), which served as the reference method in the present study, is noteworthy. However, its effective application depends on certain assumptions (such as the normal distribution of residuals, a lack of autocorrelation, and homoscedasticity), which are not necessary for the rest of the methods. On the other hand, complex data mining models, especially ANNs (such as MLP and RBF), require the estimation of a large number of parameters and bear an increased risk of overfitting if the size of the dataset is relatively small. Therefore, the second predictive performance evaluation method, i.e., 10-fold cross-validation, was applied in our study, but the obtained results were mostly concurrent with the train–test split procedure. In general, larger datasets should be used in such cases to make reliable predictions.

In a study by Fatih et al. [7], in which MARS was applied to predict the ABW of eight camel breeds (the same as in the present study) based on selected morphological traits, the following performance measures were achieved on the test set: *r*—0.97; *RMSE*—12.07 kg; *SD_ratio_*—0.25; *ME*—−1.72 kg; *RAE*—0.00; *MAPE*—1.21%; *MAD*—7.97 kg; coefficient of determination (*R*^2^)—0.93; adjusted coefficient of determination (*AdjR*^2^)—0.90; *AIC*—269.11; and *AIC_C_*—284.11. It can be seen that, in general, these results were better than those obtained in our study, which may have been caused by the different set of predictors (less correlated with ABW) included in the current work.

MARS was also used by Tırınk et al. [21], who predicted the ABW of Pakistani Marecha camels from different morphological traits (e.g., shoulder height) and sex. They reported the following performance indicators: *r*—0.93; *SD_ratio_*—0.38; *ME*—−0.26 kg; *RAE*—0.01; *MAPE*—6.98%; *MAD*—15.84 kg; *R*^2^—0.86; *AdjR*^2^—0.84; *AIC*—358.77; and *AIC_C_*—359.51. Again, these results were generally better than (*r*, *SD_ratio_*, *ME*, *R*^2^) or comparable (*RAE*, *MAPE*, *MAD*, and *AIC*) to those obtained in the present study. Recently, Asadzadeh et al. [9] applied four machine learning methods, i.e., the Bayesian-regularized neural network, extreme learning, random forest, support vector machines, and MLR, to body weight prediction in Iranian dromedary camels. The reported *R*^2^ values (0.95, 0.93, 0.95, 0.94, and 0.93, respectively) were, in general, higher than those in the present work.

Simple and multiple linear regression was used for predicting the body weight of Algerian camels based on chest girth, neck length, wither height, body length, and tail length [38]. The R^2^ values for the different age groups ranged between 0.87 and 0.98 and were higher than those obtained for the data mining models and MLR in the present study. Also, Boujenane [10] estimated the ABW of different camel breeds using six mathematical equations, including wither height, chest girth, and hump girth. The reported model performance measures were as follows: *R*^2^ from 0.68 to 0.87, *RMSE* from 18.32 to 28.39 kg, and *ME* from −129.8 to 24.7 kg. Hence, the *R*^2^ values exceeded those in the present work (except for MLP), whereas *RMSE* and *ME* had a wider range. Ihuthia et al. [1] predicted the live body weight of camel calves (up to one year of age) from shoulder height, heart girth, and abdominal girth. The *R*^2^ values for the simple and multiple linear regression models ranged from 0.17 to 0.92 and were more diverse than those in our study.

The most important predictor of ABW for all the tested models was BREED, which is an important source of variation in this trait [39]. This relationship was also confirmed by significant differences in all the variables (including ABW) among the breeds. Meghelli et al. [38] reported that breed factor significantly affected the live weight, neck length, neck girth, body length, wither height, and chest girth of two Algerian camel breeds (Steppe and Sahraoui). It was also the second most important predictor of ABW for the MARS model developed by Fatih et al. [7], which included 13 out of 25 initially considered variables (birth weight, face width, face length, and hump width, among others). MILK was ranked second among the most influential factors affecting ABW in the present study. According to Knoess [24,25], the ratio of average daily milk yield to camel’s body weight was 1.86%. Also, the daily milk production of the dromedary, ranging between 15 and 40 L, represented from 3.3% to 8.9% of its body weight. In their later study, Knoess et al. [26] stated that the mean daily milk yield (18.68 L) of seven dromedaries constituted 3.26% of their body weight. Finally, Khanna [40] observed that average daily milk yield during different stages of lactation ranged between 1.9% and 2.5% of a camels’ body weight.

The third and fourth most important predictors of ABW in the present study were AGEB and dry period (DP). The association between these traits was described by Kamal El-Den et al. [41] and Zaky et al. [42], who reported that she-camels were mated for the first parity at an appropriate body weight of 350–400 kg. The fifth and six most influential variables were age at puberty (AGEP) and lactation length (LACT). Wilson [43] described the relationship between AGEP and body weight; i.e., the effect of appropriate body weight (among other factors such as nutrition, photoperiod, temperature, and water availability) on the onset of sexual activity. Moreover, the attainment of puberty is influenced by the overall growth and weight of an animal, which are, in turn, affected by nutrition [44]. Animals with a higher plane of nutrition begin puberty earlier, and the influence of body weight on puberty is even stronger than that of animal age [43,45]. In addition, the higher the percentage of ABW at puberty is, the lower the AGEP is. In Tunisia [46,47], 83% of females are conceived at 32 months of age at a body weight of 64% of that of the adult animal. In another study [48], all females weighing more than 250 kg showed follicular activity earlier and were successfully bred at two years of age. These results are in line with the negative correlation between AGEP and ABW observed in our study. A similar relationship exists between AGEB and ABW [45]. The importance of the remaining predictors differed depending on the model used.

In the other studies on body weight prediction in camels, various sets of variables were utilized. For instance, the most influential factors affecting the ABW of Pakistani Marecha camels predicted by MARS included sex, shoulder height, hump height, and chest girth [21], while body length, whither-to-pin length, and chest girth constituted the best predictors for the four machine learning and MLR models applied by Asadzadeh et al. [9] in Iran. In a study by Boujenane [10], who estimated body weight using different mathematical equations, chest and hump girth were most strongly correlated with the true and predicted weight values, whereas wither height showed the weakest correlation. In the body weight assessment of camel calves using MLR [1], the correlation coefficients for heart girth, abdominal girth, and shoulder height were 0.93, 0.96, and 0.43, respectively. Consequently, these traits accounted for 87.0%, 91.4%, and 17.2% of body weight variation, respectively.

Finally, Fatih et al. [7] performed the hierarchical cluster analysis of camel breeds, but their results were different from ours. However, it should be emphasized that our analysis included a different set of variables. Therefore, the second cluster analysis of individual animals (not breeds) was carried out for comparison, and the clusters mostly corresponded to the eight investigated camel breeds. In general, cluster analysis is a valuable unsupervised learning technique for grouping objects (cases) into meaningful categories by measuring the similarity between features [49]. It plays a pivotal role in identifying the hidden patterns in unlabeled data [50,51]. Building separate models for each cluster often yields better results than a single model, as it accounts for subgroup-specific properties. Clustering also simplifies complex datasets, eliminating noise or irrelevant features, thus reducing dimensionality and highlighting representative cases [52,53,54]. It can also be used to engineer features for predictive models in order to improve their performance, e.g., by clustering the original sparse data (coded as binary vectors) [49]. Finally, clustering serves as a diagnostic tool for predictive modeling. Distinct groups within a dataset may indicate the underlying trends exploitable by a model, whereas overlapping clusters might suggest the need of feature engineering or domain-specific adjustments [55]. In a biological context, the cluster analysis of local breeds based on their live weight and morphological measures allows for differentiating among subpopulations, with relevant implications for breed conservation and selection programs. The limited genetic flow among such populations revealed in this way may be indicative of a substantial genetic structure, which increases conservation complexity [56]. Cluster analysis can also be utilized to investigate farmer preferences for breeding objective traits. It provides an understanding of how preferences are distributed across respondents and how they can be optimally grouped together, especially as preferences are likely to differ, even within industry segments [57].

Finally, one important limitation of the present study should be emphasized, i.e., the small dataset for model training and testing. The performance indicators for the data mining models, especially MLP and RBF, could probably be higher after training on a larger dataset. Therefore, the preliminary results obtained in this work should be verified in the future.

## 5. Conclusions

In conclusion, the different models (MLR, CART, CHAID, EXCHAID, MARS, MLP, and RBF) applied to ABW prediction in camels based on BREED and productive and reproductive parameters were moderately accurate in this regard. From among them, MLP followed by MLR showed the best predictive performance compared to the other models (*R*^2^ equal to 70.0% and 67.0%, respectively). The most important predictor of ABW was BREED, with the order of the remaining variables depending on the model used. Hierarchical cluster analysis confirmed the differences existing among the breeds. Finally, an important limitation of the present study is its relatively small dataset, especially for training the ANN (MLP and RBF). Hence, the obtained preliminary results should be validated on larger datasets in the future.

## Figures and Tables

**Table 1:** Descriptive statistics for continuous variables.

Variable	Training + Validation Set (*n* = 100)		Testing Set (*n* = 35)		Whole Dataset (*n* = 135)	
	Mean	SD	Mean	SD	Mean	SD
HAIR, kg	2.21	0.34	2.34	0.42	2.24	0.37
MILK, L	1744.21	210.68	1723.94	176.74	1738.96	201.99
LACT, days	459.33	95.67	480.14	92.15	464.73	94.87
AGEP, days	1216.69	101.08	1186.83	122.93	1208.95	107.49
AGEB, days	1455.12	118.55	1443.14	157.97	1452.01	129.39
GEST, days	391.11	19.4	385.26	14.12	389.59	18.31
DP, days	342.76	34.58	332.4	36.08	340.07	35.14
CI, days	757.95	38.51	765.6	32.46	759.93	37.07
ABW ^1^, kg	655.69	42.82	669.06	38.48	659.16	42.01

HAIR = hair production; MILK = milk yield per lactation; LACT = lactation length; AGEP = age at puberty; AGEB = age at first breeding; GEST = gestation period; DP = dry period; CI = calving interval; SD = standard deviation. ^1^ Predicted variable.

**Table 2:** Categories of nominal predictor (camel breed; BREED).

Breed	Training + Validation Set (*n* = 100)		Testing Set (*n* = 35)		Whole Dataset (*n* = 135)	
	*n*	%	*n*	%	*n*	%
Bravhi	13	13.00	4	11.43	17	12.59
Kachi	14	14.00	3	8.57	17	12.59
Pishin	12	12.00	5	14.29	17	12.59
Makrani	13	13.00	4	11.43	17	12.59
Kohi	16	16.00	1	2.86	17	12.59
Lassi	14	14.00	3	8.57	17	12.59
Rodbari	10	10.00	7	20.00	17	12.59
Kharani	8	8.00	8	22.86	16	11.85

**Table 3:** Pearson’s correlation coefficients between the predictors and the predicted variable.

Variable	HAIR	MILK	LACT	AGEP	AGEB	GEST	DP	CI	ABW
HAIR	1.00								
MILK	−0.10	1.00							
LACT	0.20 *	0.50 *	1.00						
AGEP	−0.44 *	0.15	−0.07	1.00					
AGEB	−0.18 *	0.03	0.22 *	0.66 *	1.00				
GEST	−0.23 *	0.08	−0.08	0.20 *	0.21 *	1.00			
DP	−0.24 *	−0.01	0.08	0.46 *	0.54 *	0.18 *	1.00		
CI	−0.10	−0.30 *	−0.28 *	−0.44 *	−0.52 *	0.09	−0.24 *	1.00	
ABW	−0.06	0.40 *	0.25 *	−0.34 *	−0.48 *	−0.18 *	−0.23 *	0.26 *	1.00

* Significant at *p* < 0.05.

**Table 4:** Predictor and predicted variables according to breed.

Var.	Breed	Bravhi	Kachi	Pishin	Makrani	Kohi	Lassi	Rodbari	Kharani
	*n*	17	17	17	17	17	17	17	16
HAIR	Mean	2.35 ^cd^	2.21 ^bc^	1.72 ^a^	2.00 ^ab^	2.29 ^cd^	2.20 ^bc^	2.78 ^d^	2.38 ^cd^
	SD	0.12	0.21	0.21	0.13	0.18	0.34	0.32	0.23
MILK	Mean	1658.35 ^ab^	2049.29 ^d^	1714.94 ^bc^	1929.00 ^d^	1837.82 ^cd^	1335.18 ^a^	1703.00 ^bc^	1680.63 ^ab^
	SD	32.42	17.39	33.84	37.23	15.92	17.03	10.95	23.73
LACT	Mean	579.18 ^d^	542.06 ^cd^	368.00 ^ab^	526.76 ^cd^	377.06 ^ab^	318.00 ^a^	461.94 ^bc^	549.81 ^d^
	SD	16.76	16.71	17.68	11.52	31.80	19.20	4.62	20.10
AGEP	Mean	1282.59 ^c^	1278.41 ^c^	1231.00 ^c^	1203.65 ^bc^	1297.53 ^c^	1232.35 ^c^	1024.65 ^a^	1115.94 ^ab^
	SD	28.84	83.78	67.49	62.59	92.80	54.10	55.74	27.46
AGEB	Mean	1557.71 ^d^	1529.65 ^cd^	1325.76 ^ab^	1453.88 ^bc^	1522.76 ^cd^	1513.76 ^cd^	1206.88 ^a^	1509.06 ^cd^
	SD	52.75	83.83	69.31	29.81	76.33	29.56	55.53	58.88
GEST	Mean	379.88 ^a^	394.24 ^abc^	388.12 ^abc^	405.53 ^c^	389.41 ^abc^	399.35 ^bc^	377.00 ^a^	382.81 ^ab^
	SD	3.89	26.17	13.46	15.84	15.08	23.06	9.23	11.36
DP	Mean	369.12 ^d^	313.65 ^ab^	326.76 ^abc^	354.59 ^cd^	384.88 ^d^	338.18 ^bc^	282.76 ^a^	351.31 ^cd^
	SD	12.83	24.57	19.56	17.71	23.63	16.36	9.40	12.92
CI	Mean	719.82 ^a^	727.00 ^ab^	795.18 ^d^	776.53 ^cd^	726.82 ^abc^	778.24 ^d^	787.65 ^d^	768.75 ^bcd^
	SD	19.48	20.57	13.74	15.91	48.23	17.27	6.84	27.44
ABW	Mean	641.12 ^ab^	648.12 ^ab^	703.65 ^c^	676.06 ^bc^	645.41 ^ab^	584.47 ^a^	687.24 ^c^	688.94 ^c^
	SD	15.65	25.49	18.11	28.06	15.33	32.31	15.29	23.60

Var = variable. ^a,b,c,d^ Values within a row with different superscripts differ significantly at *p* < 0.05.

**Table 5:** The predictive performance for the six models on the test set (*n* = 35).

Criterion	MLR	CART	CHAID	EXCHAID	MARS	MLP	RBF	Mean
*r*	0.82 *	0.76 *	0.79 *	0.79 *	0.78 *	0.84 *	0.79 *	0.81 *
*R* ^2^	0.67	0.56	0.61	0.61	0.59	0.70	0.60	0.65
*RMSE*	21.71	25.23	23.74	23.74	24.42	20.86	24.51	22.57
*SD_ratio_*	0.57	0.66	0.63	0.63	0.64	0.54	0.65	0.60
*ME*	−1.09	−2.02	0.61	0.61	1.32	4.28	−1.54	0.55
*RAE*	0.00	0.00	0.00	0.00	0.00	0.00	0.00	0.00
*MAPE*	2.47	2.95	2.71	2.71	2.85	2.44	2.82	2.58
*MAD*	16.51	19.72	18.21	18.21	19.12	16.45	18.69	17.28
*AIC*	219.45	229.96	225.70	225.70	227.68	216.65	227.93	222.16
*AICc*	219.82	230.34	226.08	226.08	228.05	217.03	228.31	222.53

* Significant at *p* < 0.05.

**Table 6:** The relative predictor importance for the six models (rank 1—the most important; rank 9—the least important; 100%—the most important; 0%—the least important).

Predictor		MLR	CART	CHAID	EXCHAID	MARS	MLP	RBF	Mean
BREED	rank	1	1	1	1	2	1	1	1
	%	100.00	100.00	100.00	100.00	50.00	100.00	100.00	91.67
MILK	rank	6	2	3	3	2	2	3	3
	%	77.94	79.40	39.54	39.54	50.00	30.99	22.84	50.12
AGEB	rank	3	3	5	5	1	4	9	4
	%	92.18	67.48	31.89	31.89	100.00	29.97	14.10	55.94
DP	rank	2	8	2	2	2	8	6	4
	%	90.41	26.46	40.78	40.78	50.00	28.75	14.85	41.88
LACT	rank	8	4	8	8	3	3	2	5
	%	58.16	62.86	17.14	17.14	0.00	30.71	23.72	32.10
AGEP	rank	5	7	4	4	2	9	5	5
	%	49.98	34.06	37.54	37.54	50.00	28.42	15.50	35.91
CI	rank	4	5	6	6	3	5	8	5
	%	84.00	42.38	30.63	30.63	0.00	29.27	14.22	33.42
HAIR	rank	9	6	7	7	3	7	7	7
	%	48.55	35.70	30.35	30.35	0.00	28.81	14.45	26.31
GEST	rank	7	9	9	9	3	6	4	7
	%	15.57	13.37	11.29	11.29	0.00	29.21	16.19	14.27

## Data Availability

Data available on request from the authors.

## Abbreviations

The following abbreviations are used in this manuscript:

MLR	Multiple linear regression
CART	Classification and regression tree
CHAID	Chi-square automatic interaction detection
EXCHAID	Exhaustive chi-square automatic interaction detection
MARS	Multivariate adaptive regression spline
MLP	Multilayer perceptron
RBF	Radial basis function network
HAIR	Hair production
MILK	Milk yield per lactation
LACT	Lactation length
AGEP	Age at puberty
AGEB	Age at first breeding
GEST	Gestation period
CI	Calving interval
ABW	Adult body weight
DP	Dry period
